# The importance of curve severity, type and instrumentation strategy in the surgical correction of adolescent idiopathic scoliosis: an in silico clinical trial on 64 cases

**DOI:** 10.1038/s41598-021-81319-z

**Published:** 2021-01-19

**Authors:** Fabio Galbusera, Andrea Cina, Matteo Panico, Tito Bassani

**Affiliations:** 1 Laboratory of Biological Structures Mechanics, IRCCS Istituto Ortopedico Galeazzi, via Galeazzi 4, 20161 Milan, Italy; 2 Department of Chemistry, Materials and Chemical Engineering “Giulio Natta”, Politecnico di Milano, Milan, Italy

**Keywords:** Biomedical engineering, Medical research, Paediatric research, Translational research

## Abstract

Adolescent idiopathic scoliosis is a three-dimensional deformity of the spine which is frequently corrected with the implantation of instrumentation with generally good or excellent clinical results; mechanical post-operative complications such as implant loosening and breakage are however relatively frequent. The rate of complications is associated with a lack of consensus about the surgical decision-making process; choices about the instrumentation length, the anchoring implants and the degree of correction are indeed mostly based on personal views and previous experience of the surgeon. In this work, we performed an in silico clinical trial on a large number of subjects in order to clarify which factors have the highest importance in determining the risk of complications by quantitatively analysing the mechanical stresses and loads in the instrumentation after the correction maneuvers. The results of the simulations highlighted the fundamental role of the curve severity, also in its three-dimensional aspect, and of the instrumentation strategy, whereas the length of the fixation had a lower importance.

## Introduction

Adolescent idiopathic scoliosis (AIS) is a three-dimensional deformity of the spine appearing during puberty and showing largely variable severity and curve patterns among the affected individuals^1^. In select cases, AIS can be treated conservatively by means of physiotherapy and/or bracing, with the aim of stopping the progression of the curves^2^. In general, less severe curves tend to have a lower risk of progression^3^; for this reason, surgical correction is considered in skeletally immature subjects in which the Cobb angle in the coronal plane exceeds a certain threshold, typically 50°^4,5^. Nevertheless, surgical treatment is nowadays also considered in less severe cases, down to Cobb angles of 35°^4^, and leads in most cases to very good or excellent clinical and cosmetic results^6^. Indeed, the technologies and implants currently available to the surgeons allow for a safe reduction of the coronal and three-dimensional deformities while preserving a correct sagittal alignment^6^.

The use of instrumentation, however, is associated with peri-operative and post-operative complications related with the challenging biomechanical environment, especially after the correction of severe deformities. Bartley et al. described cases of intra-operative loss of fixation to bone, disassembly of implants, and broken rods while performing aggressive correction manoeuvres^7^. In addition, Weiss and Goodall described a varied but high rate of post-operative complications such as mechanical failure of the instrumentation, pseudoarthrosis, screw loosening, loss of correction, and several others^8–11^. Delayed complications associated with the posterior rods are relatively common; Flynn et al. observed radiographic adverse events, including rod breakage, loss of correction as well as proximal screw pull-out in 6.8% of patients treated with posterior instrumentation for Lenke type 1 AIS^12^.

As a matter of fact, although a considerable amount of studies about the surgical treatment of AIS has been published, the surgical decision-making, which is aimed at maximizing the clinical outcome while minimizing the risk of complications, still involves several open questions, even when restricting the options to posterior fixation only. The planning of the instrumentation length, the choice of the anchoring devices and their implantation patterns, i.e. skipping some levels or using more implants on the convex side, as well as the selection of the rod material and diameter remain indeed matters of debate. This lack of consensus has been highlighted in papers in which experienced spine deformity surgeons were asked to select the instrumentation strategy for some cases of AIS^13–15^, in which a large variability in the pre-operative plannings proposed by the different experts was found, with obvious implications in terms of clinical outcome and occurrence of mechanical complications such as implant loosening and breakage. The authors attributed this variability to personal views and preferences for the targets for correction, to previous experience, to the inter-rater variability in the classification systems used as a support for the planning^1,16^ as well as to the lack of standardized strategies and rational rules based on biomechanical studies, which are currently relatively limited^14^.

The aim of this paper is to provide a large set of biomechanical data to support the definition of standardized strategies and rules for the pre-operative planning of AIS surgery. By simulating the correction of a large number of AIS cases covering a wide range of severity scenarios, curve types as well as possible instrumentation strategies, we aimed at predicting the most important parameters determining high stresses and mechanical loads in the instrumentation, which were assumed to be associated with post-operative mechanical complications.

## Materials and methods

### Patients and imaging

This computational study was carried out in accordance with relevant guidelines and regulations. Fully anonymized biplanar radiographs acquired with the EOS Imaging System (EOS Imaging, Paris, France) were available for 192 subjects with age ranging from 10 to 18 years old from a previous clinical trial^17^ which was approved by the Ethics Committee of Ospedale San Raffaele, Milan (protocol EOSCC2013) and during which subject assent and parental permission to use anonymized data in successive studies were given by signing an informed consent. Among those, the 64 subjects having Cobb angle in the coronal plane larger than 35° were included in the study. The resulting cohort had a Cobb angles in the range between 36.8° and 84.6°, with a median value of 54.6°. The T4–T12 kyphosis ranged between 0.2° and 72.5° with a median of 25.9°, whereas the range of the L1–L5 lordosis was 3.2°–77.3° with a median value of 44.1°. The location of 55 relevant landmarks, 32 for each vertebra in the T1–L5 region and 11 for the pelvis, were automatically extracted from the three-dimensional reconstruction of the spinal anatomy obtained by means of sterEOS proprietary software (EOS Imaging, Paris, France) through a reverse-engineering approach. The extent of all scoliotic curves, as well as the curve type based on the Lenke classification^1^, were then automatically determined by means of purposely developed Python scripts based on the location of the landmarks.

### Finite element modeling

**Figure 1:**
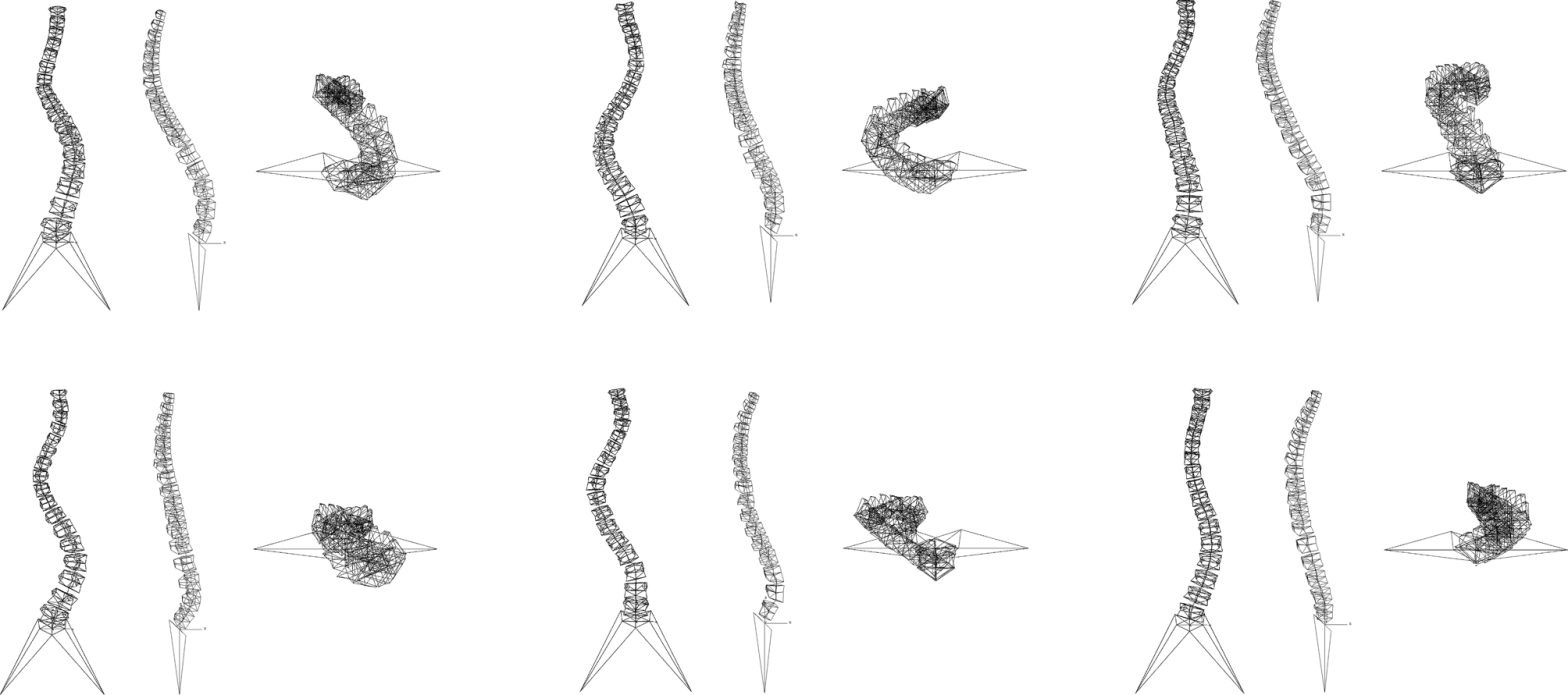
Six representative finite element models of the thoracolumbar spine of scoliotic subjects. For each model, the coronal, sagittal and axial views are shown.

Simple finite element models of the T1-sacrum region of all spines aimed at simulating deformity correction with posterior instrumentation were automatically built (Fig. 1). The models consisted exclusively of beam elements and simulated each vertebra and the pelvis as rigid structures, i.e. not capable of deforming under mechanical load, and intervertebral discs as beam elements with cross-sectional areas fitting the size of the adjacent endplates and fixed material properties (elastic modulus of 6 MPa, Poisson ratio of 0.45) mimicking the global behavior of the intervertebral disc^18^. Facet joints and ligaments were not included in the models. The posterior rods were modeled as beam elements with a circular section, a diameter of 5.5 mm and the material properties of titanium (elastic modulus of 110 GPa, Poisson ratio of 0.3).

### Target configurations and instrumentation

A large number of correction and instrumentation scenarios were simulated for each spine model. In total, 16 target configurations, i.e. spine alignments to be obtained after deformity correction, were defined. The unaltered sagittal curvature with respect to the pre-correction condition was considered as baseline configuration, i.e. as reference for the generation of the models in which sagittal corrections were implemented. With respect to the baseline, increases of lumbar lordosis and thoracic kyphosis up to 30° in steps of 10°, in all the possible 15 permutations (0 degree of lordosis and 10 degree of kyphosis, 0° and 20°, etc.) were simulated. The target Cobb angle of all scoliotic curves in the coronal plane as well as the vertebral rotations in the transverse plane were set to zero in all 16 configurations.

**Figure 2:**
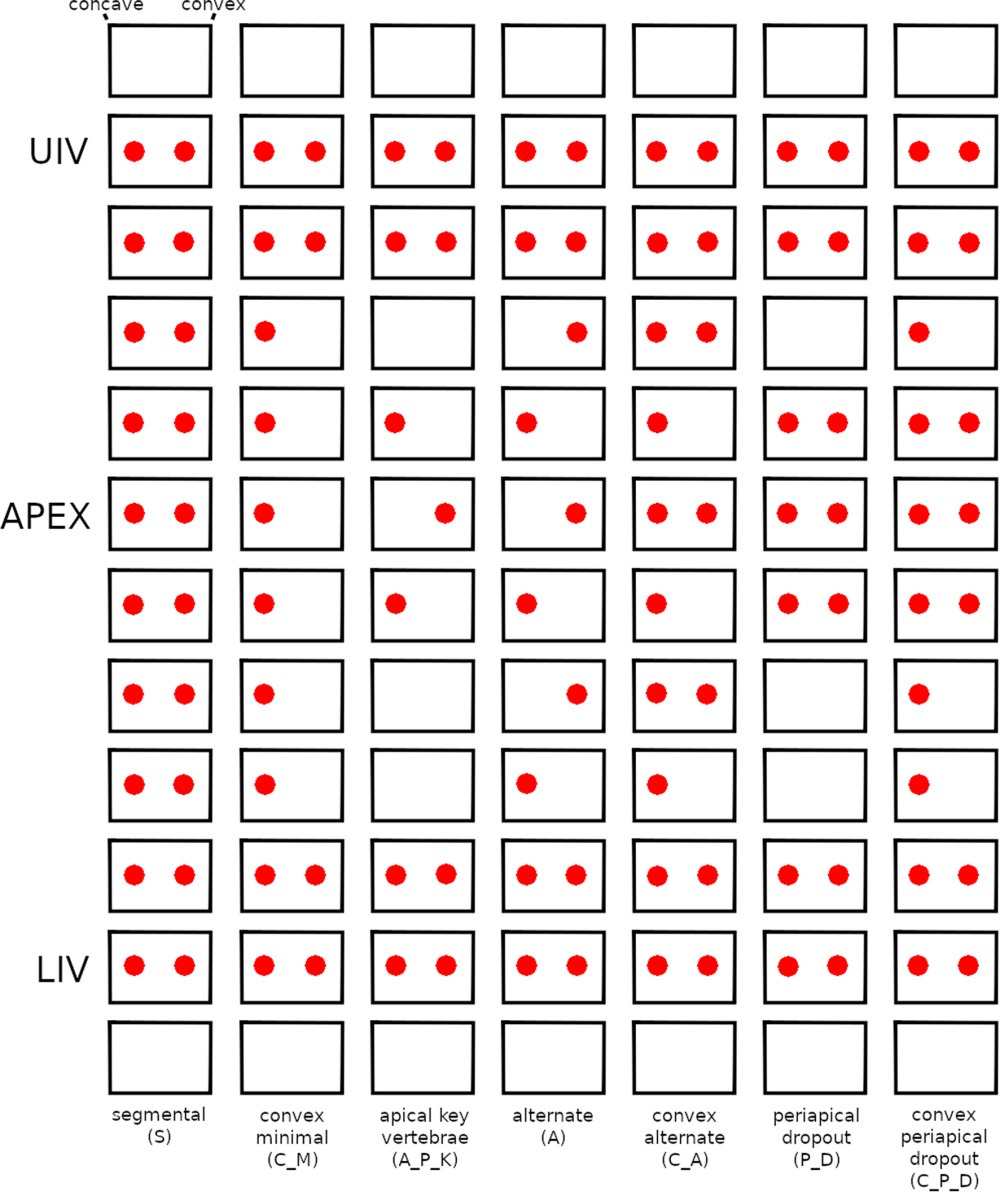
The seven screw patterns investigated: segmental (S), convex minimal (C_M), apical key vertebrae (A_P_K), alternate (A), convex alternate (C_A), periapical dropout (P_D), convex periapical dropout (C_P_D). *UIV* upper instrumented vertebra, *APEX* apex of the curve with the largest Cobb angle, *LIV*: lower instrumented vertebra.

Seven screw patterns were simulated: segmental, convex minimal, apical key vertebra, alternate, convex alternate, periapical dropout, convex periapical dropout (Fig. 2)^19^. For each pattern, three different locations of the upper instrumented vertebra (UIV) were considered, namely 4, 5, and 6 vertebrae cranially to the apex of the major curve; similarly, the lower instrumented vertebra (LIV) was simulated 4, 5, and 6 vertebrae caudally to the apex. The possible range for the instrumentation was restricted to the T1–S1 region. In total, 63 simulations were conducted for each target configuration, resulting in 1008 simulations for each subject, except for cases in which the location of the apex determined positions of UIV and/or LIV outside of the allowed instrumentation range. For each of the simulations, the ideal contour of the posterior rods was geometrically calculated based on the position of the pedicles in the target configuration (i.e. Cobb angles equal to zero for all the coronal curves and prescribed thoracic kyphosis and lumbar lordosis as described above) and on the instrumentation length.

Surgical correction of the deformity by means of polyaxial screws and posterior rods was modeled using an established approach^20,21^, appropriately adapted to the beam models used in the current study. In brief, in the first loading step the nodes corresponding to the center of the pedicles were simultaneously displaced so that their final position matched the position of the pedicles in the target configuration; in the second step, these boundary conditions were released and kinematic couplings between the nodes of the pedicles and the closest nodes on the posterior rods were defined. As a result, the elastic energy due to the deformation imposed in the first step, not constrained anymore by the nodal boundary conditions at the pedicles, determined the strains and stresses in the instrumentation which were the subject of the present study. Rotational degrees of freedom were not constrained at the screw–rod junction.

### Data analysis and statistics

Data analysis and visualization were performed by means of custom Python scripts, taking advantage of free SciPy (https://www.scipy.org/) and Seaborn (https://seaborn.pydata.org/) software. The metrics which were used for the quantitative comparison between the different curve severity, types, and instrumentation strategies were: (1) the maximal stress in the posterior rods after deformity correction; (2) the maximal magnitude of the force between pedicle screws and posterior rods, measured as the load transmitted by the kinematic couplings; (3) the craniocaudal position of the maximal rod stress with respect to the apex of the curve with the largest Cobb angle in the coronal plane; (4) the craniocaudal position of the maximal screw–rod interaction force with respect to the apex. The features which were considered as possible predictors for the metrics were: Lenke type^1^; largest Cobb angle in the coronal plane; screw pattern; apical vertebra of the curve with the largest Cobb angle; rotation in the transverse plane of the apical vertebra; upper and lower vertebrae of the curve with the largest Cobb angle; side of the convexity (levoscoliosis or dextroscoliosis); UIV; LIV; change in the lumbar lordosis in the target configuration with respect to the pre-correction configuration; change in the thoracic kyphosis.

The importance of these features in determining the maximal rod stress and screw–rod interaction force was determined by means of a gradient boosting regression model^22,23^. In addition, after checking the non-normality of the distributions, Kruskal–Wallis tests were performed to assess the differences in instrumentation stresses and forces due to Lenke type, Cobb angle in the coronal plane and apical rotation, as well as for the different screw patterns and instrumentation lengths. In case of significance, Wilcoxon rank-sum tests with Bonferroni correction to account for multiple comparisons were then performed to detect the differences between the groups. To this purpose, the scoliotic spines were stratified by Cobb angle and by apical rotation with bins having width of 5°.

## Results

**Figure 3:**
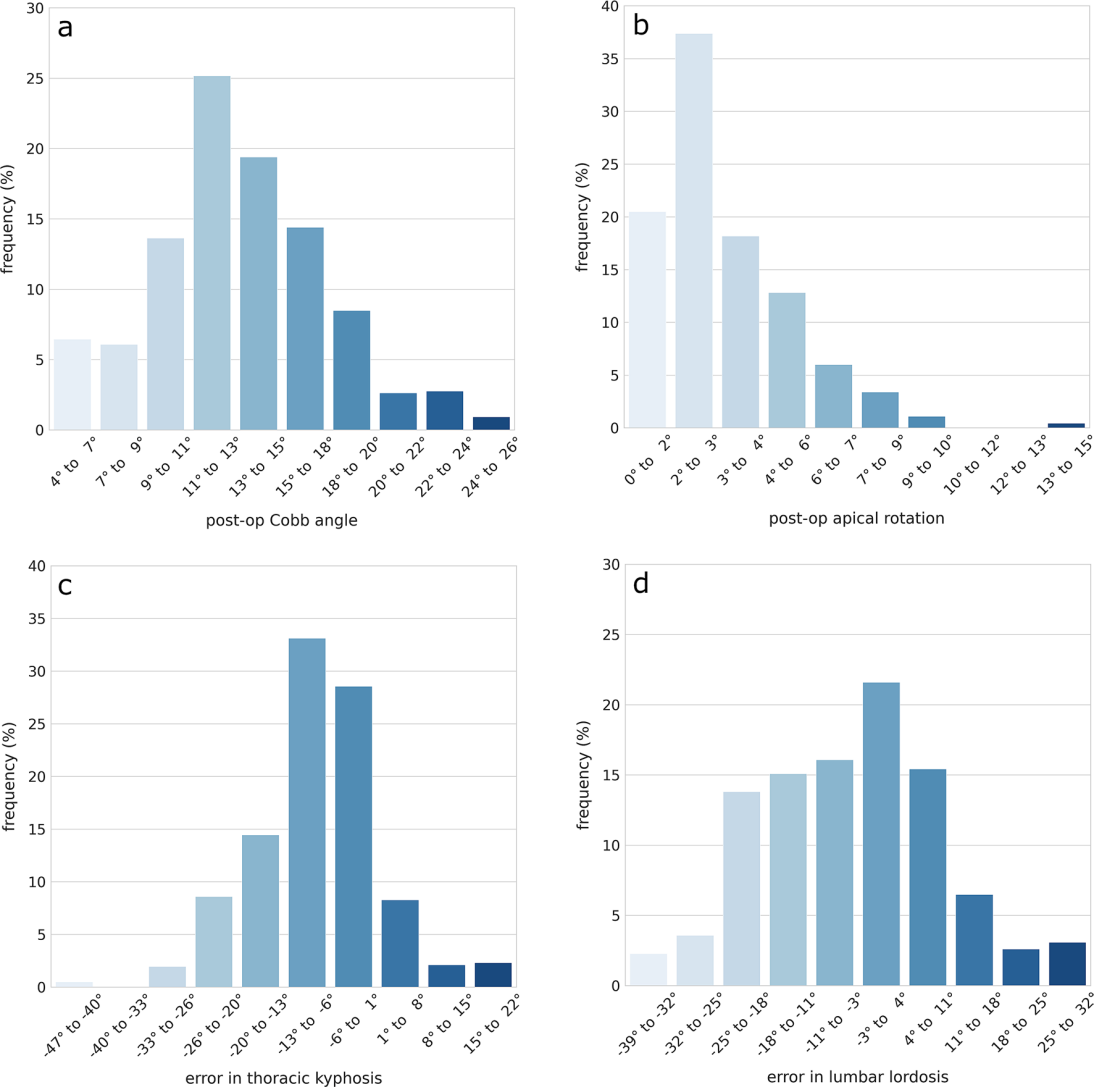
Distributions of the post-operative major Cobb angle in the coronal plane (**a**), of the post-operative transverse rotation of the apical vertebra (**b**), of the difference between the target thoracic kyphosis (**c**) and lumbar lordosis (**d**) and the predicted values. In (**c**,**d**), negative values should be intended as undercorrections, and positive values as overcorrections.

The simulated surgical procedures allowed for a major but not complete reduction of the coronal Cobb angle of the scoliotic curves and of the transverse rotation of the apical vertebra, which showed residual values up to 25.9° and 14.5°, respectively, and median values of 13.2° and 2.6° (Fig. 3). The differences between the target sagittal parameters and the achieved ones showed larger ranges (Fig. 3), the extremes of which were associated with short instrumentations.

**Figure 4:**
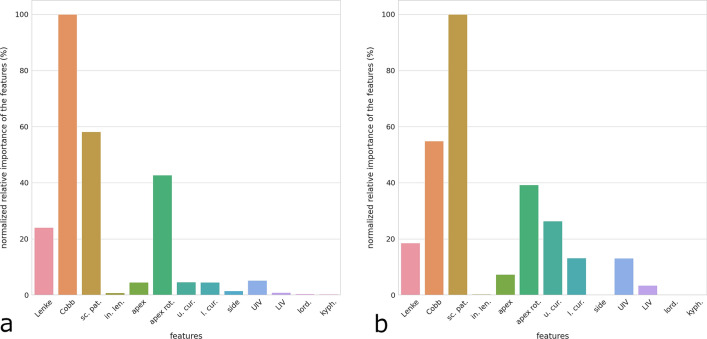
Relative importance of the features in determining the maximal stress in the rods (**a**) and the maximal force at the screw–rod interface (**b**). *Lenke* Lenke type, *Cobb* Cobb angle in the coronal plane of the largest curve, *sc. pat*. screw pattern, *in. len*. instrumentation length, *apex* location of the apex of the largest curve, *apex rot*. rotation of the apex in the transverse plane, *u. cur*. upper end vertebra of the deformity, *l. cur*. lower end vertebra of the deformity, *UIV* upper instrumented vertebra, *LIV* lower instrumented vertebra, *lord*. change in lumbar lordosis with respect to the pre-operative condition, *kyph*. change in thoracic kyphosis.

The most important features for the determination of the maximal rod stress were, in descending order, Cobb angle, screw pattern, apical rotation, and Lenke type, whereas all other factors had importance lower than 5% with respect to the Cobb angle (Fig. 4a). Regarding the maximal screw–rod force, the most relevant feature was the screw pattern, followed by Cobb angle and apical rotation; upper and lower end vertebrae of the largest curve, UIV and Lenke type had also a non-negligible importance (Fig. 4b).

**Figure 5:**
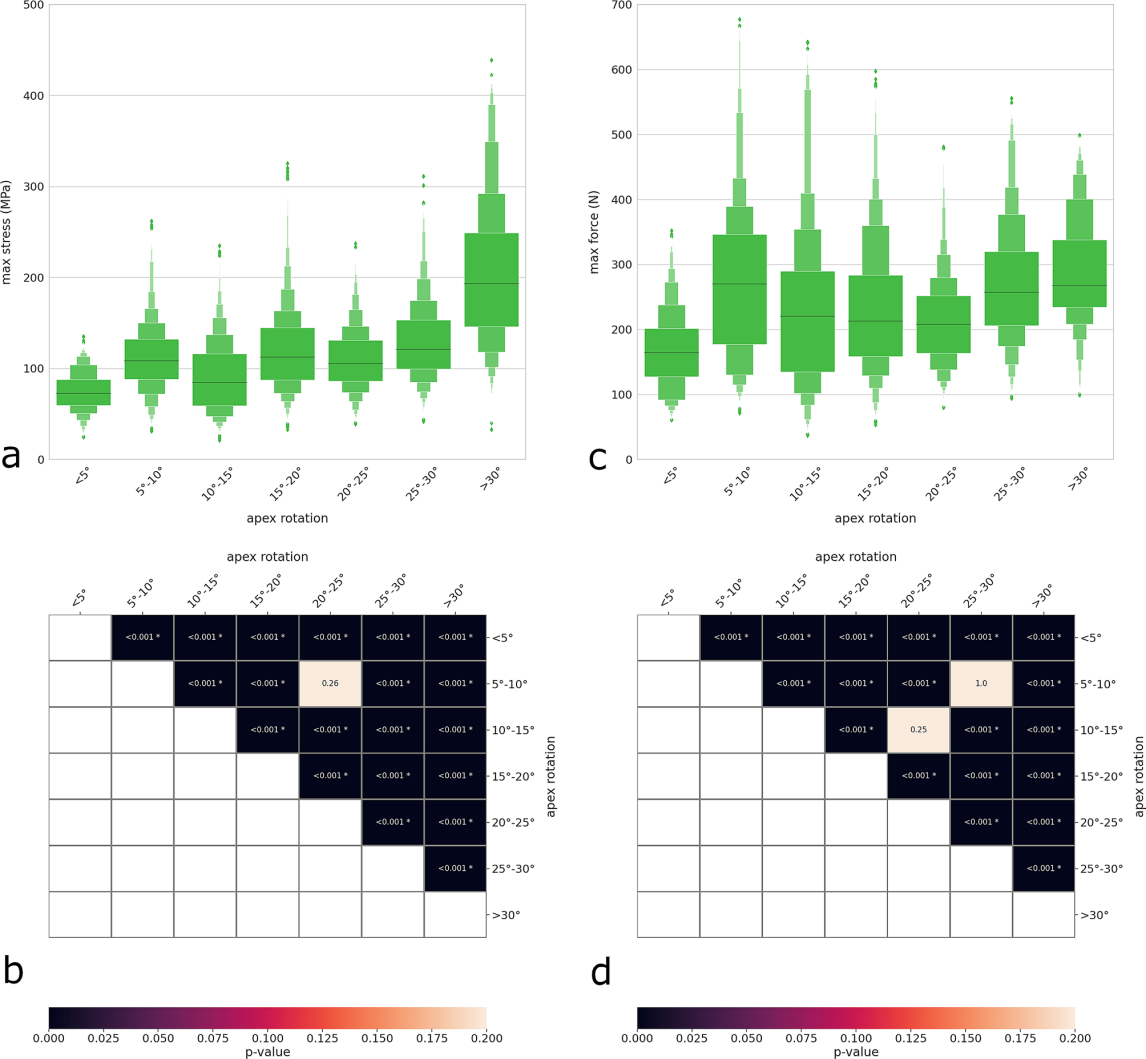
Distribution plots showing the maximal rod stress (**a**) and maximal screw–rod force (**c**) with respect to the transverse rotation of the apex of the largest curve. Heatmaps and p values of the statistical comparisons between groups regarding the maximal rod stress (**b**) and the maximal screw–rod force (**d**).

Increasing apical rotation determined increases in both maximal rod stress and screw–rod force (Fig. 5a,c). The median stresses and forces for the highest apical rotations (> 30°) showed respectively a 2.8- and 1.6-fold increase with respect to those for rotations lower than 5°. Strong statistical significance was found for all comparisons between groups, with a few exceptions (Fig. 5b,d).

**Figure 6:**
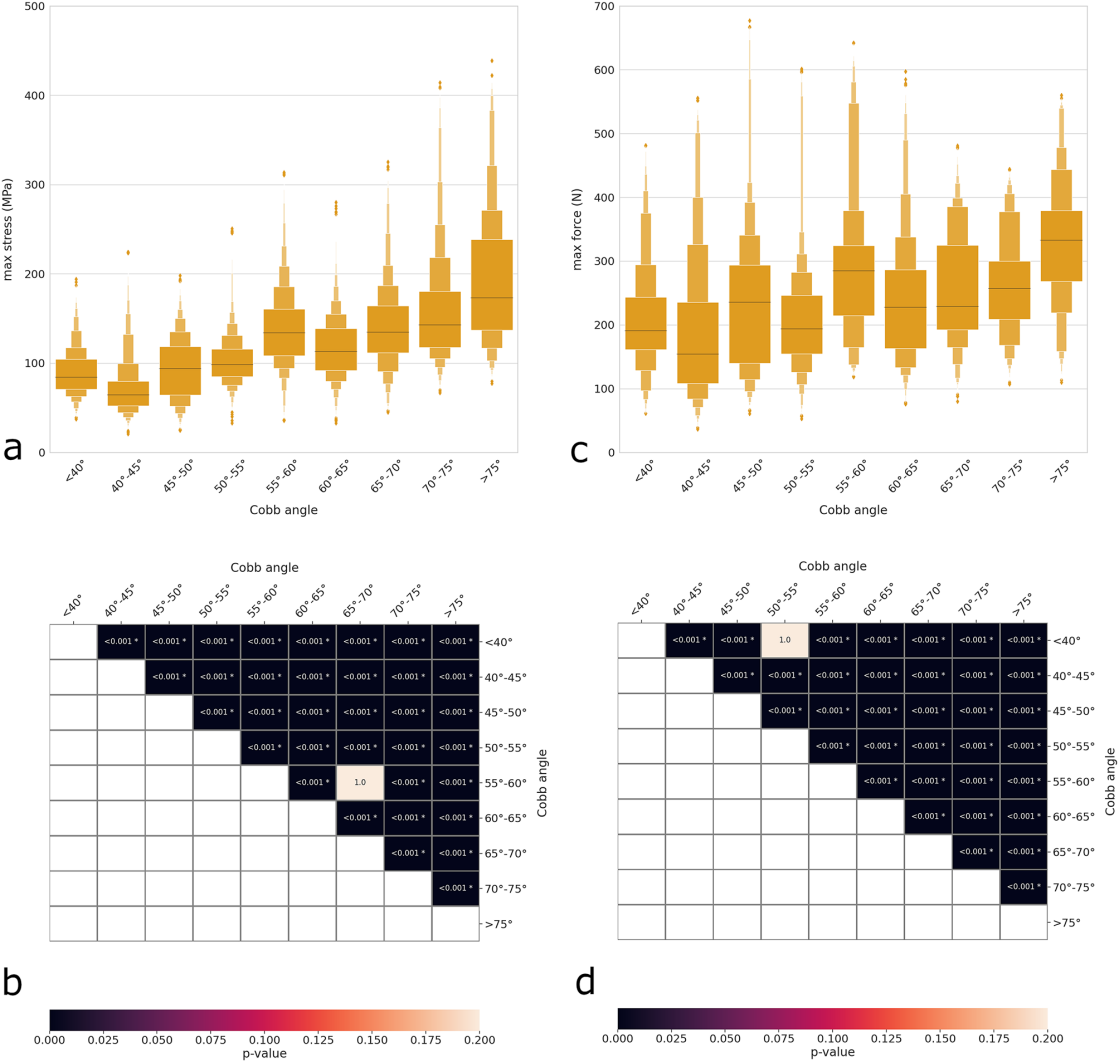
Distribution plots showing the maximal rod stress (**a**) and maximal screw–rod force (**c**) with respect to the Cobb angle of the largest curve in the coronal plane. Heatmaps and p values of the statistical comparisons between groups regarding the maximal rod stress (**b**) and the maximal screw–rod force (**d**).

A similar pattern was found for the maximal Cobb angle in the coronal plane, which was strongly associated with increases in both rod stress and screw–rod force (Fig. 6a,c); in this case, increases in Cobb angle determined higher changes in maximal rod stresses (twofold increase for angles greater than 75° with respect to those lower than 40°) than those in maximal screw–rod forces. Statistical significance was found for the large majority of comparisons between groups (Fig. 6b,d), with a few exceptions.

**Figure 7:**
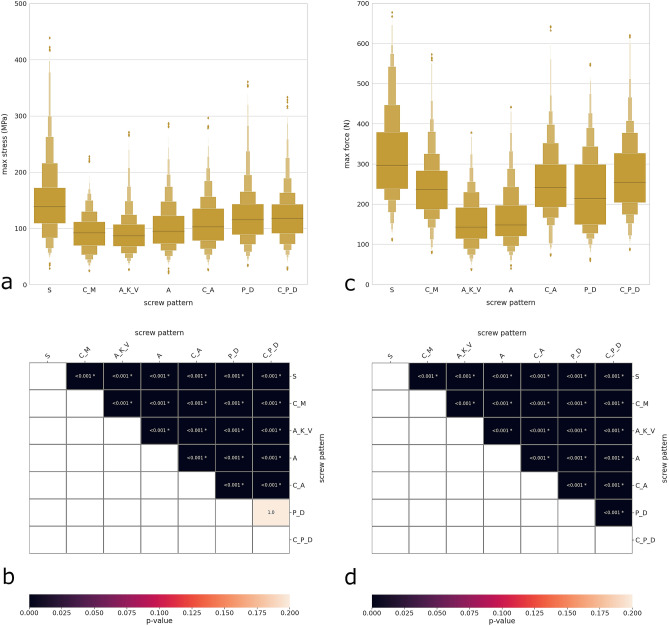
Distribution plots showing the maximal rod stress (**a**) and maximal screw–rod force (**c**) with respect to the screw pattern. Heatmaps and p values of the statistical comparisons between the different screw patterns regarding the maximal rod stress (**b**) and the maximal screw–rod force (**d**). *S* segmental, *C_M* convex minimal, *A_K_V* apical key vertebrae, *A* alternate, *C_A* convex alternate, *P_D* periapical dropout, *C_P_D* convex periapical dropout.

**Figure 8:**
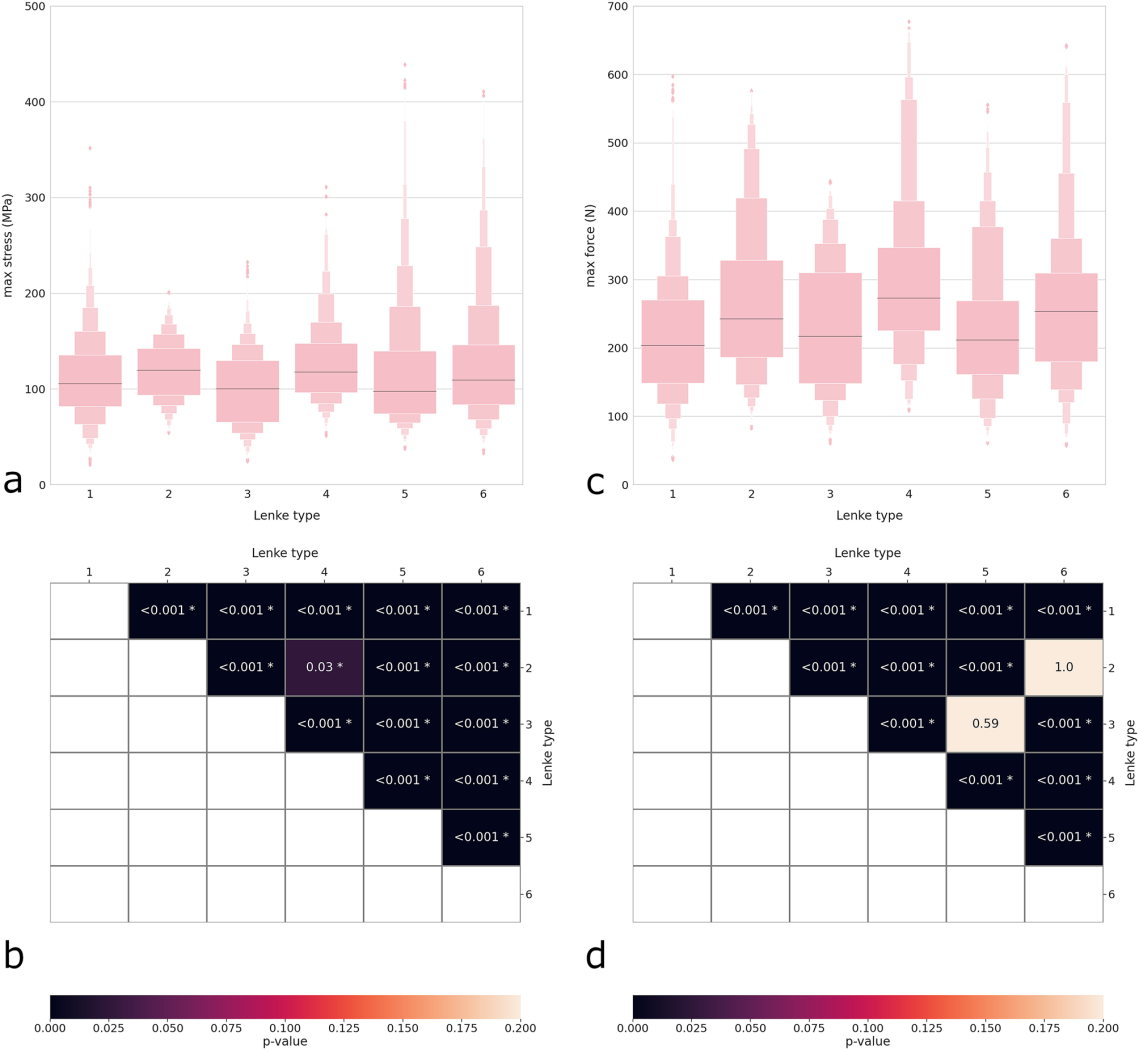
Distribution plots showing the maximal rod stress (**a**) and maximal screw–rod force (**c**) with respect to the Lenke type. Heatmaps and p values of the statistical comparisons between the different Lenke types regarding the maximal rod stress (**b**) and the maximal screw–rod force (**d**).

The effect of screw pattern and Lenke type on maximal stresses and forces was more subtle in comparison with apical rotation and Cobb angle (Figs. 7, 8). In general, the segmental screw pattern was associated with higher stresses and forces. The other patterns determined rather similar maximal stresses (Fig. 7a), although the large number of simulations allowed for strong statistical significance even if the differences in median values were low (Fig. 7c). Regarding the maximal screw–rod forces (Fig. 7b), the differences between screw patterns emerged more clearly. The segmental pattern determined the highest forces (median value of 295 N), followed by convex minimal, convex alternate and convex periapical dropout (median values of 240–250 N). Apical key vertebrae and alternate patterns induced the lowest values of maximal forces (140–150 N).

The Lenke types which were associated with highest stresses and forces were 2, 4 and 6; nevertheless, the differences in median values with respect to the other types were relatively low (Fig. 8a,b). Due to the high number of samples, the small differences were sufficient to determine strong statistical significance for most group comparisons (Fig. 8c,d).

**Figure 9:**
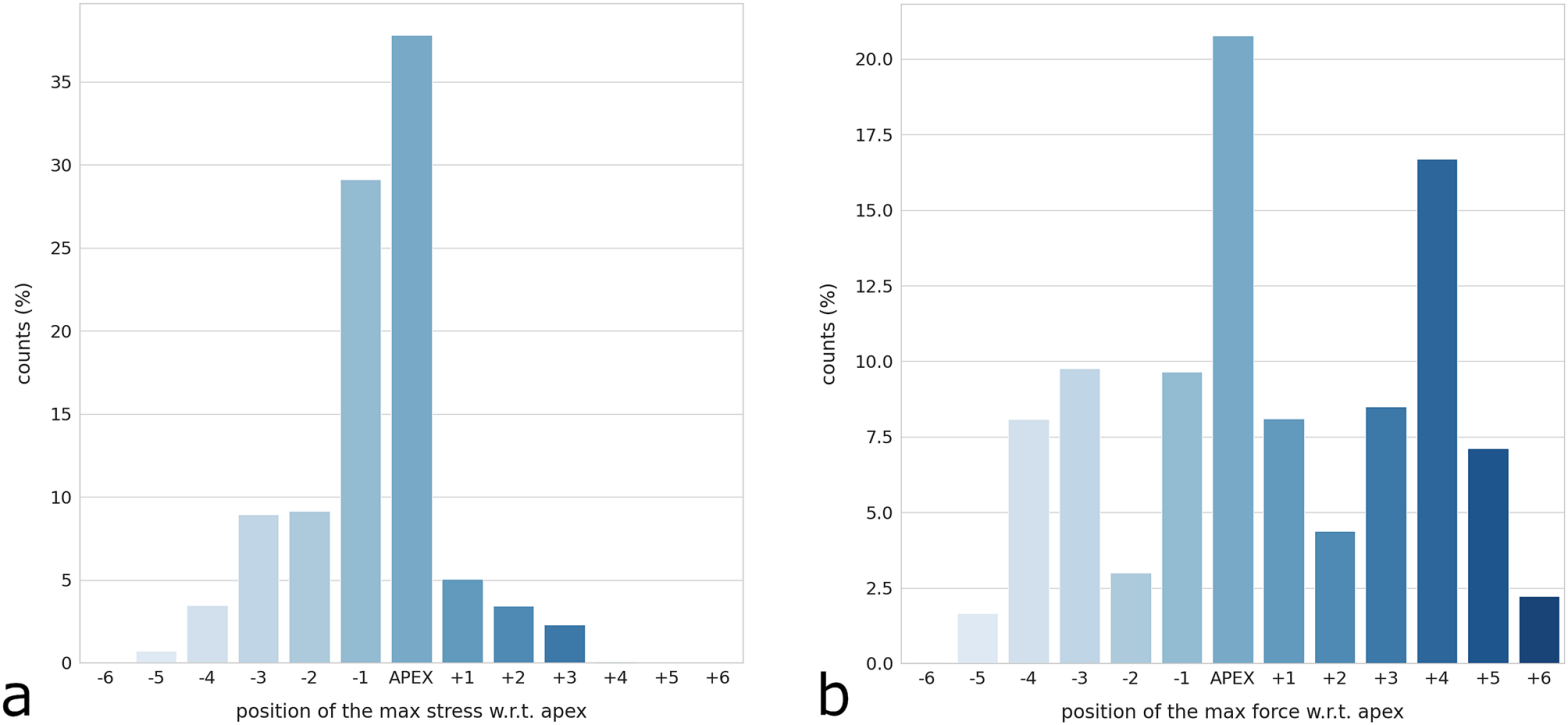
Frequency plot of the position of the maximal rod stress (**a**) and of the maximal screw–rod force (**b**) with respect to the apex of the largest curve. Negative values (− 1, − 2, etc.) indicate the vertebrae above the apex, i.e. proceeding in the cranial direction, whereas positive values (+ 1, + 2, etc.) indicate the vertebrae caudal to the apex.

The positions of the maximal rod stress (Fig. 9a) and of the maximal screw–rod force (Fig. 9b) with respect to the apex of the major curve showed distinct behaviors. For both metrics, the position with the highest occurrence was the apical vertebra itself; however, whereas the distribution of the location of the maximal rod stress resembled a bell curve centered on the apex, the maximal screw–rod forces were distributed across the whole instrumentation length, including its proximal ends.

## Discussion

In this paper, we estimated the relevance of several features describing the pre-operative shape of the spine, the targets for the surgical correction of the deformity as well as the surgical instrumentation in determining the mechanical stresses and loads in the instrumentation due to the correction maneuvers. In general, the most important predictors of high stresses were the rotation in the transverse place of the apical vertebra, the maximal Cobb angle in the coronal plane, and the screw pattern. Other predictors, such as Lenke type and UIV, had also a non-negligible importance. Such findings were obtained by means of a large simulation campaign on a high number of subjects and considering several instrumentation strategies and target configurations, with a general approach more influenced by big data analytics rather than by the consolidated techniques used in biomechanics. As a matter of fact, the present study constitutes an in silico clinical trial, a novel paradigm for the assessment of medical devices and surgical techniques which is already having a heavy impact on scientific studies and the medical industry^24,25^. To our knowledge, this study constitutes the first in silico clinical trial about AIS correction.

While models replicating the anatomy and biomechanics of specific patients, i.e. patient-specific models, have been documented in numerous papers, in silico clinical trials are a relatively novel development that still needs to gain a wide adoption by the scientific community. Despite the similarities, the scope of the two paradigms is different: while patient-specific models aim at investigating one specific patient, condition, pathology or treatment to gain better knowledge or to guide the clinical decisions about that specific case, in silico trials aim at a higher level of evidence by providing statistical significance data about some quantities (efficacy, safety, etc.) in the investigated population^24,25^. In other words, in the case of patient-specific models the outputs concern the case under investigation while for in silico clinical trials refer to the population object of the study.

As mentioned above, the simulations highlighted the importance of some features in determining instrumentation stresses and loads, which in turn have a direct impact on the risk of complications such as loosening and breakage. The mechanical relevance of the severity of the deformity, and in particular of the apical rotation, is well described in the scientific literature. The analysis of the vertebral rotation in coronal radiographic projections has been documented for decades^26–28^, and although the pathogenesis of scoliosis remains unclear^29,30^, the potential role of vertebral rotation and of the three-dimensional aspect of the deformity has been highlighted in this respect^27,31^. As a matter of fact, the determination of the apical rotation has become a fundamental step in the current workflow for the pre-operative planning of AIS correction^32,33^, especially after the introduction of precise three-dimensional reconstructions of the vertebral anatomy based on biplanar radiographic systems^32,34^. The current results highlighted the critical importance of the apical rotation, and demonstrated that its surgical management is one of key issues, perhaps the most important at all, to be considered in the pre-operative planning phase.

Another fundamental feature was the screw pattern, which was especially critical in determining the screw–rod interaction force. In a study about 279 patients with Lenke 1 AIS^19^, i.e. a main thoracic curve, the authors analyzed how the different screw patterns as well as the instrumentation length influence the degree of achieved correction, and showed that the screws in the apical concavity were the most strongly associated with curve correction. On the contrary, the global implant density had a more limited impact, consistently with other studies^35–37^. In other words, low-density scenarios had a good correction potential if they included screws in the concave side of the apical region. Although in the current study we did not directly investigate the capability of correction of the various screw patterns, we did not find large differences among the patterns in which the apex and the adjacent vertebrae were completely instrumented on the concave side (convex minimal, convex alternate, periapical dropout, convex periapical dropout), whereas other patterns with lower density determined lower stresses and loads (alternate, apical key vertebrae). Assuming that the higher loads are associated with a better capability of correction, our simulations confirmed the importance of instrumenting the apical and immediately adjacent vertebrae on the concave side.

The biomechanical analysis of the surgical correction of AIS remains largely unexplored, and any quantitative comparison and validation of the current data with analogous results has thus necessarily a limited extent. To our knowledge, no experimental data about the loads and stresses during the correction of AIS with posterior instrumentation has been published. Klöckner et al.^38^ described a forceps which could be used to measure the tensile load in a single-rod ventral correction implant, but provided no quantitative loading data. Fairhurst et al.^39^ measured and reported correction forces generally in line with the present results (88–1019 N), but also focused on anterior correction techniques. Lou et al.^40^ measured correction forces between 22 and 57 N in seven patients in which AIS was corrected by means of the derotation manoeuvre and posterior fixation, which are in general lower than our average predicted value of 74 N. However, the literature study mostly focused on the description and validation of the measurement device, whereas the clinical data collection was relatively small and documented in a limited manner. Pankowski et al. experimentally measured the torque-to-failure and angle-to-failure during the derotation manoeuvre in whole cadavers, finding average values of 73 Nm and 45° and failures most commonly located in the apical area^41^. Cheng et al. compared failure loads after derotation with different instrumentation configurations, and concluded that linked constructs provided higher failure loads than single pedicle screws^42^.

In comparison with in vivo studies, previous numerical models provide a larger reference set for comparing and validating the current results. In a pioneering study based on a rigid-body model of spine deformities and their surgical correction which would constitute the basis of a large series of successive papers, a research group at École Polytechnique de Montréal published values of the reaction forces at the implant-vertebra links, which had peak values at around 1000 N^43^. In a successive study of the same group, Wang et al. reported bone-screw forces up to approximately 500 N in polyaxial screws in 10 AIS patients with an average value of 141 N^44^. Although it should be noted that the force values reported in this study refer to the screw–rod interface rather than the bone-implant connection and direct comparisons should be considered only in semi-quantitative, approximate terms, we predicted maximal and average correction forces of 677 and 74 N respectively, which are in general consistent with the literature results; our lower average force could be partially attributed to the different location in which the forces were calculated as well as to the fact that Wang and colleagues simulated only the segmental screw pattern, which is associated with higher forces (Fig. 7c). Indeed, the same research group reported correction forces on average 50% higher in instrumentations with high implant density in comparison with those with less pedicle screws^45,46^; in the present study, we found an average force of 96 N when restricting the analysis to the segmental configurations. In a recent study, Clin et al. simulated a total of 150 clinical AIS correction scenarios on five patients and predicted average forces of 99 and 96 N for the high-density and low-density scenarios respectively^47^. Salmingo et al. reported values of correction forces in similar ranges (average of 125 N, maximum of 375 N) in 6 patients in which AIS was corrected with high-density screw patterns^48^.

The Canadian research group also investigated other biomechanical quantities less directly comparable with the current data. Robitaille et al. simulated various instrumentation strategies in five AIS patients by means of a biomechanical model including rigid elements as well as deformable structures^49^. The simulations were not aimed at the prediction of the instrumentation stresses, but at calculating a “cost function” describing the quality of the achieved correction in three-dimensional terms. The authors found differences between the target coronal alignments and the predicted ones up to 17°, well comparable with the present results (Fig. 3a). The approach was refined by La Barbera et al., who defined an optimization function describing the achieved correction and the residual mobility and used it to investigate various instrumentation and correction strategies in a pediatric patient with a thoracic curve^50^. Similar models were used in other studies, which focused on the evaluation of correction targets on the instrumentation strategy^51,52^. Several other papers by various research groups described the simulation of AIS correction in specific patients or in a small set of subjects by means of finite element models^53–58^, but to our knowledge no one reported data which can be directly compared with the present ones.

The simple beam-based modeling approach is a major limitation of this study, and should be accounted for in light of the need for the full automatization of model generation, execution and postprocessing. Indeed, automated scripts proved to be a necessity for the management of such a large population and of a high number of target configurations and instrumentation strategies; as a matter of fact, automatization is a key issue in large-scale in silico trials^25^. Vertebrae were considered as rigid; intervertebral discs were modeled with a single beam element with material properties not accounting for the alterations due to the deformity^59^; facet joints and ligaments were neglected; the presence of the rib cage was not considered as well. Although similar approaches have been employed in previous studies^60–62^, these simplifications definitely had an impact on the accuracy and validity of the results, as shown by numerous papers in which the importance of these anatomical structures was highlighted^63–67^.

Furthermore, all material properties were assigned on a patient-independent basis, whereas it is well known that different patients exhibit different degrees of spinal flexibility^68^, especially in the case of deformities^59^, and that the mechanical properties of spinal tissues have a major role in determining the resulting spine biomechanics^69–71^ and consequently on the preoperative planning. In this study, we nevertheless decided to employ this simplified approach in order to better focus on the importance of the spinal curvature and of the instrumentation strategy, leaving out the patient-dependent stiffness which could have acted as a confounding factor. Besides, the assessment of patient-specific properties in spinal deformity patients remains challenging and prone to uncertainties, and requires in any case additional radiographic images^57^ which were not available for the present set of patients. Furthermore, the boundary conditions used to model the rod engagement allowed free rotations, which correctly mimicked the effect of polyaxial screws during correction but not the lock achieved after tightening the set screws. Finally, the algorithmic choice of the upper and lower instrumented vertebrae may have led to the simulation of a number of non-realistic or clinically non-acceptable instrumentation strategies, resulting in unsatisfactory corrections and possibly affecting the statistical analysis.

Although a thorough comparison of the results with previous numerical and in vivo studies was performed, an extensive validation of the models could not be achieved due to the lack of experimental biomechanical data directly investigating the effect of curvature and instrumentation strategies in AIS correction. This aspect is especially critical if in silico clinical trials are intended to be used as medical devices, i.e. to draw decisions impacting the clinical decisions for specific patients or to prove the validity of implants or surgical techniques for regulatory purposes. As a matter of fact, the models described in this paper as well as other AIS correction models reported in the literature would likely not fulfil the critera described in the ASME standard V V 40 about the credibility of computational models^72^, which requires among other things the quantification of uncertainties and the validation against high-quality in vivo or in vitro comparators, not available in this field as mentioned above. However, it should be noted that the present modelling framework was not intended to be used as a medical device but was rather aimed at providing a better understanding of the relevance of some parameters on the biomechanical outcome of the surgery, with no direct influence on specific patients or implants.

It should be noted that some of the limitations discussed above are shared with several available models of scoliosis correction, which also include rigid or beam elements and have a variable degree of sophistication^43,44,46,53^. With respect to the rigid-body models developed by the Montréal group, we did not account for the specific correction maneuvers, but we simply simultaneously imposed displacement boundary conditions to replicate, in a simplified way, the final corrected configuration. Despite all these limitations, the present dataset constitutes a highly valuable resource, especially considering that its size largely exceeds, in terms of both number of patients and surgical strategies, any biomechanical study about AIS correction previously published. Besides, it should be noted that the flexible simulation framework here presented would allow for a straightforward integration of functional loads applied after the deformity correction, such as compressive loads replicating standing, trunk flexion, carrying loads etc.

In conclusion, the fully automated simulation framework here described, which was employed on a large population of subjects with different curve types and severities, demonstrated that apical rotation, Cobb angle in the coronal plane and screw pattern are the most important predictors of high stresses and loads in the instrumentation after AIS correction. We believe that such large campaigns of biomechanical simulations, and in silico clinical trials in general, have an enormous potential for the study of AIS and for other pathologies exhibiting a high degree of inter-subject variability.
